# Theoretical Insights into the Hydrogen Evolution Reaction on VGe_2_N_4_ and NbGe_2_N_4_ Monolayers

**DOI:** 10.1021/acsomega.1c06730

**Published:** 2022-02-24

**Authors:** Mihir Ranjan Sahoo, Avijeet Ray, Nirpendra Singh

**Affiliations:** † Harish-Chandra Research Institute, Prayagraj 211019, India; ‡ Department of Physics, Indian Institute of Technology Roorkee, Roorkee 247667, India; ^§^Department of Physics and ^∥^Center for Catalysis and Separation (CeCaS), Khalifa University of Science and Technology, Abu Dhabi 127788, United Arab Emirates

## Abstract

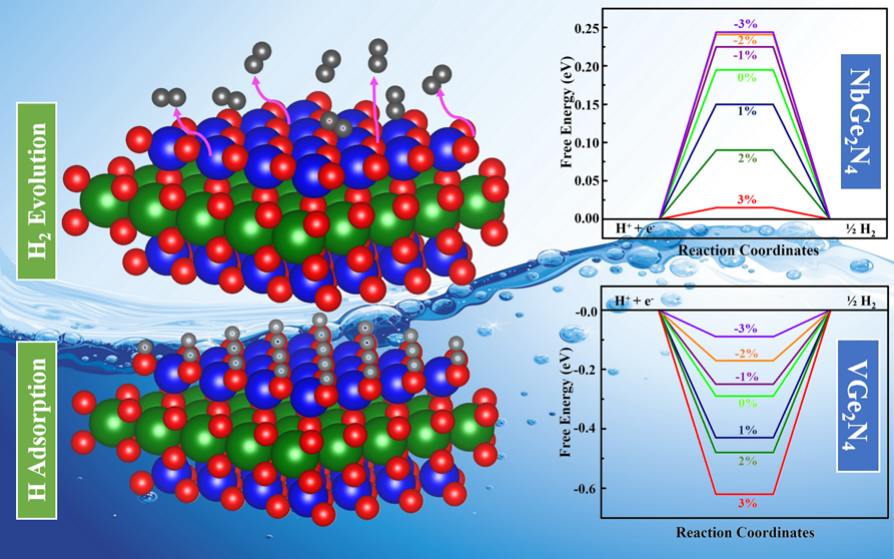

Catalytically active sites at the basal plane of two-dimensional monolayers for hydrogen evolution reaction (HER) are important for the mass production of hydrogen. The structural, electronic, and catalytic properties of two-dimensional VGe_2_N_4_ and NbGe_2_N_4_ monolayers are demonstrated using the first-principles calculations. The dynamical stability is confirmed through phonon calculations, followed by computation of the electronic structure employing the hybrid functional HSE06 and PBE+*U*. Here, we introduced two strategies, strain and doping, to tune their catalytic properties toward HER. Our results show that the HER activity of VGe_2_N_4_ and NbGe_2_N_4_ monolayers are sensitive to the applied strain. A 3% tensile strain results in the adsorption Gibbs free energy (Δ*G*_H*_) of hydrogen for the NbGe_2_N_4_ monolayer of 0.015 eV, indicating better activity than Pt (−0.09 eV). At the compressive strain of 3%, the Δ*G*_H*_ value is −0.09 eV for the VGe_2_N_4_ monolayer, which is comparable to that of Pt. The exchange current density for the P doping at the N site of the NbGe_2_N_4_ monolayer makes it a promising electrocatalyst for HER (Δ*G*_H*_ = 0.11 eV). Our findings imply the great potential of the VGe_2_N_4_ and NbGe_2_N_4_ monolayers as electrocatalysts for HER activity.

## Introduction

The search for new catalytic materials to fulfill the demand for substantial energy resources and the development of alternative, efficient, cost-effective, abundant energy sources have received significant attention within the scientific community in the last few decades. The electrochemical water splitting through the hydrogen evolution reaction (HER) is an efficient method to produce hydrogen, an eco-friendly fuel with a high energy density, that is considered a promising technique to store energy from sustainable sources.^1−3^ The well-known electrocatalysts for HER are mostly noble metals like Pt and Pd and their compounds.^4−6^ However, high cost and low abundance hinder their applications in the mass production of hydrogen.^7^

In the last few decades, two-dimensional (2D) materials have been considered promising catalysts for HER because of their large surface-to-volume ratio and more active sites. Apart from that, 2D materials are excellent substrates due to their unique structural and electronic properties. The catalytic activity can be regulated through chemical modification and structural engineering.^8^ In addition, low cost, earth abundance, and ability to form various nanostructures have led to 2D materials gaining significant research attention in the field of catalysis^9−11^ and being considered as alternatives to the widely used and costly Pt-based catalysts.^12−14^ The major drawback of 2D catalysts is that the catalytically active sites are confined to only edges while the basal plane is inert.^15−17^ Although previous experimental and theoretical studies reported that the basal planes of MXenes are catalytically active sites for HER,^18−20^ their performances are inferior to those of Pt and Pt-based compounds.^21,22^ Recently, graphene-based materials,^23,24^ reduced graphene oxides,^25,26^ g-C_3_N_4_,^27^ borophene,^28^ phosphorene,^29^ monolayers of Mo_2_C,^30^ and h-B_2_O^31^ have been proposed as promising catalysts. Furthermore, the HER performance of these 2D materials can be triggered through various strategies, such as surface functionalization,^32−34^ intrinsic defect,^35,36^ doping,^37−39^ and strain engineering.^40^ Despite numerous attempts to develop efficient 2D HER catalysts, the promising materials that can be suitable for practical device applications and take the place of Pt are still far from reality. Hence, the quest for efficient 2D electrocatalysts is essential for hydrogen energy.

Since a new 2D van der Waals (2D vdW) MA_2_Z_4_ family is recently synthesized using the chemical vapor deposition method,^41^ the attention on these 2D materials is rapidly increasing due to their potential applications.^42−46^ The high carrier mobility and excellent stability make them suitable substrates. Moreover, hydrogen adsorption on the basal plane can be tuned due to the presence of the lowest unoccupied energy level, which leads to promising electrocatalysts for HER.^47^ The HER performance of 2D MoSi_2_N_4_ and WSi_2_N_4_ can be enhanced by introducing N vacancy and doping by transition metal atoms like V, Fe, Nb, Tc, and Ta.^48^ In addition, O doping at the N site and P, Fe, and Nb doping at the Si site of 2D MoSi_2_N_4_ exhibit superior HER activity.^49^ Moreover, an intercalated architecture approach is employed to predict 70 family members that are both dynamically and thermodynamically stable.^50^ The recent HER studies on the MA_2_Z_4_ class of material have been mainly focused on MoSi_2_N_4_ and WSi_2_N_4_ monolayers only, whereas other possible 2D magnetic materials of this family have not been explored. Recently, a multilevel screening of the 2D MA_2_Z_4_ family is performed by Liu et al.^47^ to explore the basal plane active catalyst for hydrogen evolution reaction; among 144 materials, they showed that compounds based on V and Nb metals with Ge and N show better performance by considering lowest occupied state energy, which ranges between −6.0 and −5.6 eV for better HER activity, as a descriptor. However, the catalytic activities of these materials, with respect to structural change by physical or chemical means, have not been discussed so far. Therefore, exploring the novel electronic and catalytic properties of other possible 2D magnetic materials (having more catalytically active sites in a basal plane) of this family with strain, defects, doping, etc. is of great interest for the fundamental research and practical applications in the field of hydrogen evolution reaction.

We present a systematic study on the structural, electronic, and catalytic properties of monolayer VGe_2_N_4_ and NbGe_2_N_4_ using the first-principles calculations. The dynamical stability is confirmed through the phonon band structure. The calculated Gibbs free energy (Δ*G*_H*_) is −0.29 eV (VGe_2_N_4_) and 0.19 eV (NbGe_2_N_4_), which are close to the ideal value (for Pt, Δ*G*_H*_ = −0.09 eV^51^). The defects (N vacancy or Ge vacancy) and doping of nonmetal atoms (B, C, O, P, and S) are introduced to examine the performance of HER activity. Our findings will guide experimentalists in designing magnetic VGe_2_N_4_ and NbGe_2_N_4_ monolayers as excellent electrocatalysts for hydrogen evolution reactions.

## Computational Methodology

The first-principles calculations are performed using density functional theory as employed in the Vienna ab initio simulation package (VASP).^52,53^ The exchange–correlation functional prescribed by Perdew, Burke, and Ernzerhof (PBE)^54^ under the generalized gradient approximation is used for the calculation of structural and electronic properties. The spin-polarized calculations of 2 × 2 × 1 supercell are performed with a Γ-centered 6 × 6 × 1 **k**-grids to sample the Brillouin zone. The kinetic energy cutoff of 500 eV is used to describe the plane-wave basis set. The monolayers are designed to maintain periodicity along the *XY*-plane with a vacuum of 20 Å along the *z*-direction to avoid the interactions between the periodic artificial images. Full relaxation of all of the atoms is allowed until a residual force of 0.001 eV/Å is reached. The Heyd, Scuseria, and Ernezerhof screened hybrid density functional (HSE06)^55^ with van der Waals correction is used to estimate the accurate band gaps of VGe_2_N_4_ and NbGe_2_N_4_ monolayers. We also performed PBE+*U* functional to account for the onsite Coulomb interaction of d electrons of V and Nb atoms. The finite displacement method^56^ is employed to calculate the phonon band structure.

The HER performance of VGe_2_N_4_ and NbGe_2_N_4_ monolayers is described by calculating the Gibbs free energy of the reaction intermediate (H*), which is defined as
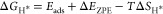
1where Δ*E*_ZPE_ and *T*Δ*S*_H*_ are the change in zero-point energy and entropy between atomic hydrogen adsorption and hydrogen in the gas phase, respectively. The contributions from the catalyst to both Δ*E*_ZPE_ and *T*Δ*S*_H*_ are very small and hence can be neglected. Thus, Δ*E*_ZPE_ can be obtained through the following equation^57^
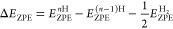
2where *E*_ZPE_^*n*H^ represents the zero-point energy of *n* hydrogen atoms adsorbed on monolayer without the contribution of the catalyst and *E*_ZPE_^H_2_^ is the zero-point energy of H_2_ molecule in the gas phase. The value of Δ*E*_ZPE_ can vary from −0.01 to 0.04 eV. The entropy of atomic hydrogen Δ*S*_H_ (≈ −1/2 Δ*S*_H_^0^), where Δ*S*_H_^0^ is the entropy of H_2_ molecule in the gas phase. The value of *TS*_H_^0^ can be considered as 0.4 eV^51^ at *T* = 300 K, which gives rise to the value of Δ*E*_ZPE_ – *T*Δ*S*_H*_ equal to 0.24 eV. Therefore, the adsorption free energy related to the HER mechanism can be considered as
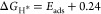
3For an ideal catalyst, the value of Δ*G*_H*_ should be zero. Here, *E*_ads_ is the hydrogen binding energy and can be defined as
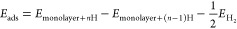
4where *E*_monolayer+*n*H_ and *E*_monolayer+(*n*–1)H_ are the total energy of the monolayer with *n* and *n* – 1 adsorbed hydrogen atoms, respectively, and *E*_H_2__ is the total energy of H_2_ molecules in the gas phase.

Based on Nørskov’s approach,^58^ the catalytic descriptor Δ*G*_H_ can be used to determine the hydrogen evolution exchange current density (*i*_0_) in the form of a volcano-shaped diagram. At pH = 0, *i*_0_ can be calculated from the following equations as
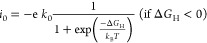
5
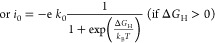
6where *k*_0_ and *k*_B_ are the rate constant and Boltzmann constant, respectively, and *k*_0_ is set to 1.

## Results and Discussion

**Figure 1:**
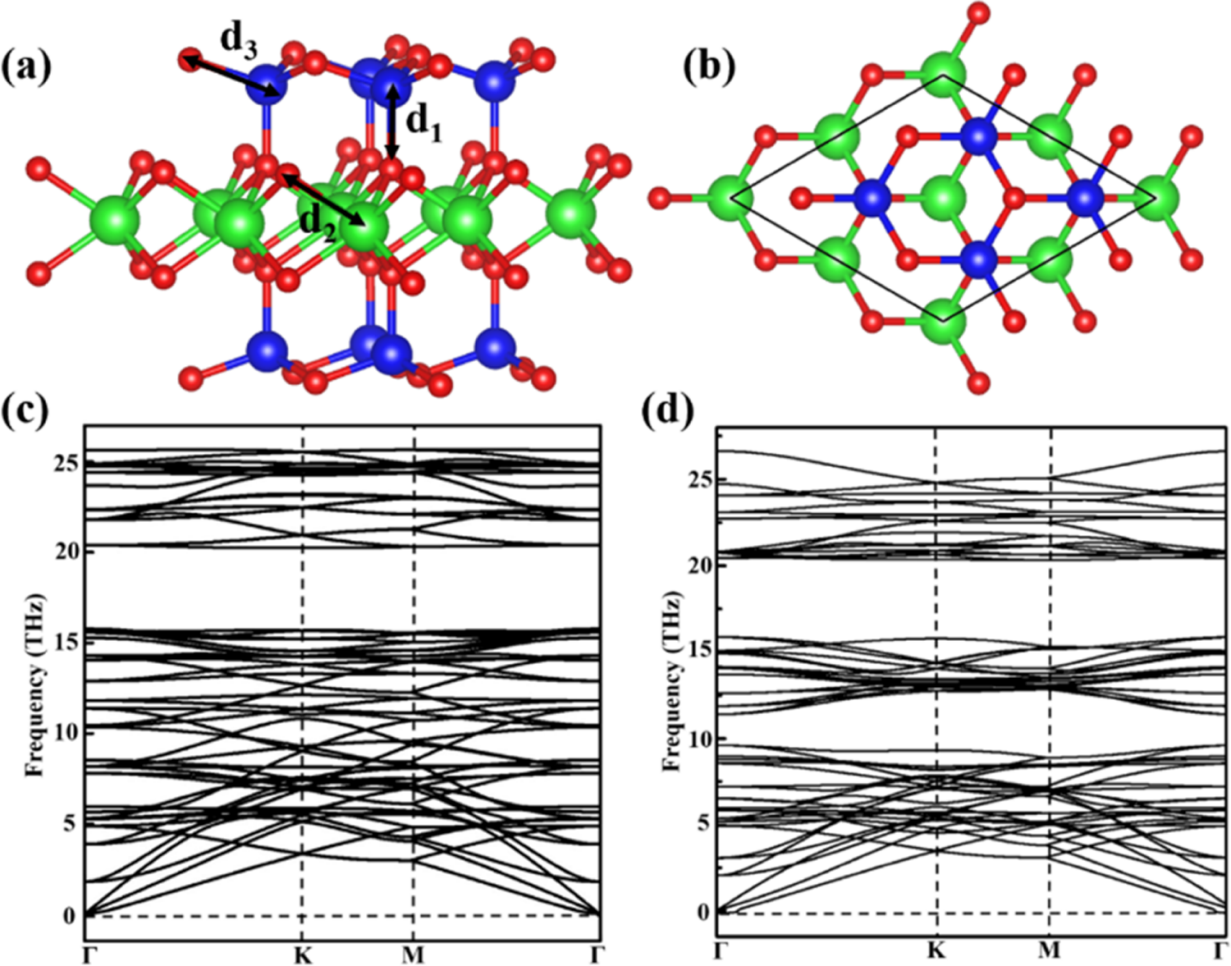
Optimized structure of 2 × 2 × 1 supercell of V/NbGeN_4_ monolayer: (a) side view and (b) top view (blue: Ge atoms, red: N atoms, green: V/Nb atoms). Phonon spectra of VGe_2_N_4_ and NbGe_2_N_4_ monolayers are shown in (c) and (d), respectively.

The in-plane optimized lattice constants of VGe_2_N_4_ and NbGe_2_N_4_ monolayers are 3.00 and 3.09 Å, respectively, consistent with the previous study.^50^ Both the monolayers exhibit hexagonal crystal structure with the space group *P*6̅*m*2 (No. 187), in which a metal layer is sandwiched between Ge and N layers in the vertical directions and stacked in the sequence of N–Ge–N–M–N–Ge–N as shown in Figure 1a,b, where M represents the heavy metal (V or Nb). It can also be viewed as an MN_2_ monolayer sandwiched between two Ge–N layers. Figure 1a shows the bond distances between interlayer Ge–N atoms, between M–N atoms (at the middle portion of the unit cell), and the intralayer Ge–N atoms (at both top and bottom portions), which are denoted by d_1_, d_2_, and d_3_, respectively. For the optimized VGe_2_N_4_ (NbGe_2_N_4_) monolayer, these values are 1.88 Å (1.87 Å), 2.06 Å (2.14 Å), and 1.85 Å (1.85 Å), respectively. The dynamical stability of the monolayers is confirmed by the positive phonon frequency in the calculated phonon band structures (Figure 1c,d).

The band structures of both the monolayers within the PBE functional are shown in Figure S1 (Supporting Information). The hybrid HSE06 functional is employed to calculate the correct band gap and gain more insight into the electronic properties because the PBE functional shows a small band gap (∼0.008 eV for VGe_2_N_4_ and ∼0.001 eV for NbGe_2_N_4_). The calculated HSE06 band gaps of VGe_2_N_4_ and NbGe_2_N_4_ monolayers are 0.69 and 0.48 eV, respectively (Figure S2 of Supporting Information). For the VGe_2_N_4_ monolayer, the valence band maximum is situated at the Γ-point, whereas the conduction band minimum is located at the *K*-point, resulting in an indirect band gap. The band gaps in the spin-up and spin-down states are 0.69 and 2.55 eV, respectively (Figure S2a). On the other hand, for the NbGe_2_N_4_ monolayer, the spin-up and spin-down band gap values are 1.46 and 2.57 eV, receptively (Figure S2b).

**Figure 2:**
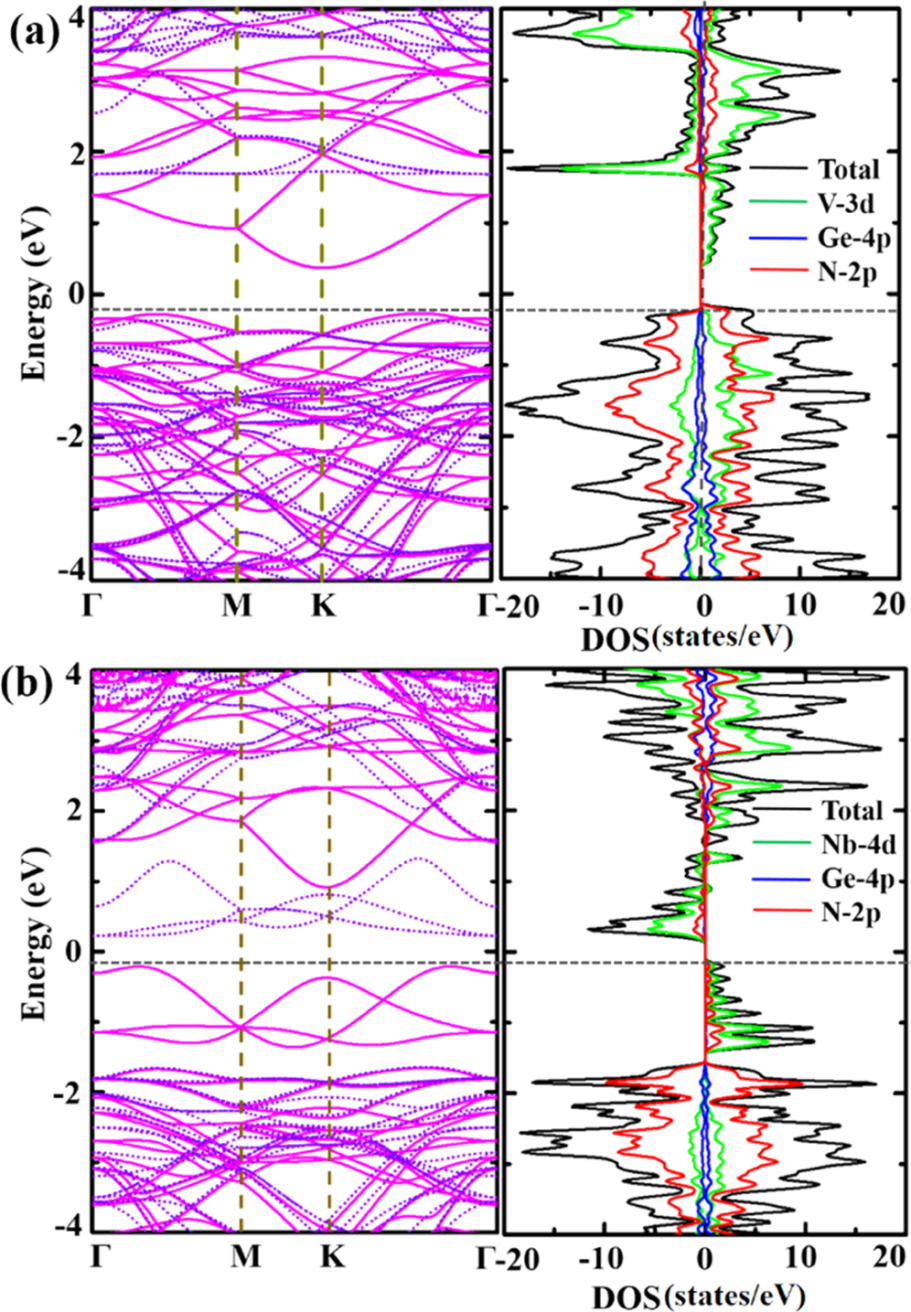
Calculated electronic band structure and project density of states of (a) VGe_2_N_4_ (*U* = 4.5) and (b) NbGe_2_N_4_ (*U* = 4). Spin-up and spin-down bands are represented by solid magenta lines and dotted purple lines, respectively. In the partial density of states, the contributions of V-3d (Nb-4d), Ge-4p, and N-2p orbitals are represented by green, blue, and red lines, respectively. The black line represents the total DOS.

The Hubbard onsite Coulomb potential *U* is used to fix the empirically over-delocalization issue of *d* electrons of V and Nb atoms. The *U* value is determined according to the HSE06 band structure. The calculated band gap at different *U* is matched with the HSE06 band gap, listed in Supporting Information (Table S1). Table S1 shows that the band gap of the VGe_2_N_4_ monolayer is found to be 0.67 eV at *U* = 4.5 eV (Figure 2a) close to the HSE06 band gap. Similarly, the band gap of the NbGe_2_N_4_ monolayer at *U* = 4 eV (Figure 2b) matches with the HSE06 band gap. Therefore, *U* = 4.5 eV (for VGe_2_N_4_) and 4 eV (for NbGe_2_N_4_) are used to further investigate the electronic and catalytic activities. The band structure and projected density of states (PDOS) of monolayers are shown in Figure 2. For the VGe_2_N_4_ monolayer, the spin-up states contribute to the conduction band minimum and are mainly dominated by V-3d states, whereas the spin-down state is away from the Fermi level. The valence band maximum is symmetrically contributed by both the spins and formed by the interaction between V-3d and N-2p states (Figure 2a), which is 0.67 eV lower than the conduction band minima. The scenario is different for the NbGe_2_N_4_ case as the valence and conduction bands near the Fermi level are contributed by spin-up and spin-down states, respectively, where a strong hybridization between Nb-4d and N-2p states is observed (Figure 2b), and are separated from each other by the gap of value 0.44 eV. In the deeper regime of the valence band, the N-2p orbitals weakly hybridized with Ge-4p and 3d/4d states of V/Nb are present.

## Hydrogen Evolution Reaction Activity

**Figure 3:**
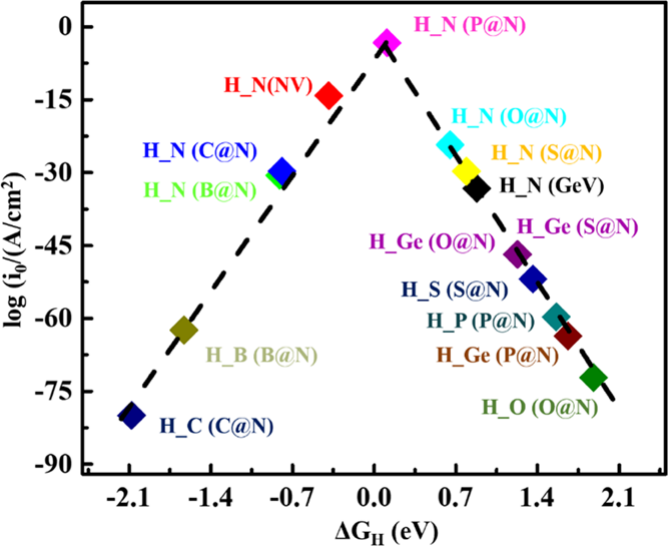
Volcano curve for the exchange current density *i*_0_ as a function of Δ*G*_H*_ for the NbGe_2_N_4_ monolayer. H_X (X = N, Ge, C, B, O, P, S) represents the structures containing an H atom adsorbed above an X atom. NV and GeV represent N vacancy and Ge vacancy defect structures, respectively. Y@N (Y = P, S, C, O, B) represent the structures with Y atom doped at the N site. (For example, H_S(S@N) represents the structure containing an H atom placed above an S atom where the S atom is placed in the place of the N site of the NbGe_2_N_4_ monolayer).

The calculated Gibbs free energy for hydrogen adsorption at N sites of VGe_2_N_4_ and NbGe_2_N_4_ monolayers are −0.29 and 0.19 eV, respectively, far from the ideal value. The strong (weak) binding of a hydrogen atom on the basal planes of VGe_2_N_4_ (NbGe_2_N_4_) monolayers prohibits them from being an efficient HER electrocatalyst. Therefore, N and Ge vacancies are introduced in the basal plane. The Δ*G*_H*_ values for N and Ge vacancies of the NbGe_2_N_4_ monolayer are −0.39 and 0.88 eV, respectively. The higher absolute binding energy value (|Δ*E*_H_|) indicates that the hydrogen evolution and adsorption are difficult in N and Ge vacancies, respectively. Later, doping of B and C atoms at N sites increases the binding energy compared to the pristine case and hinders the use of the NbGe_2_N_4_ monolayer as a promising electrocatalyst. In addition, O and S dopings at the N site show high positive Δ*G*_H*_. However, P doping at the N site causes the Δ*G*_H*_ value to be 0.11 eV, which is much lower than the previous cases, and also results in increased exchange current. Figure 3 demonstrates a volcano plot to show the best active site of the NbGe_2_N_4_ monolayer for the HER reaction. P doping at the N site represented by H_N(P@N) offers the highest exchange current density among all possible doped cases. The detailed structural and electronic properties of the P-doped NbGe_2_N_4_ monolayer are given in the Supporting Information (Figure S3–S4). The same defect and doping engineering in the VGe_2_N_4_ monolayer do not enhance the HER activity significantly. The Δ*G*_H*_ values for different doped cases are provided in the Supporting Information (Table S2).

**Figure 4:**
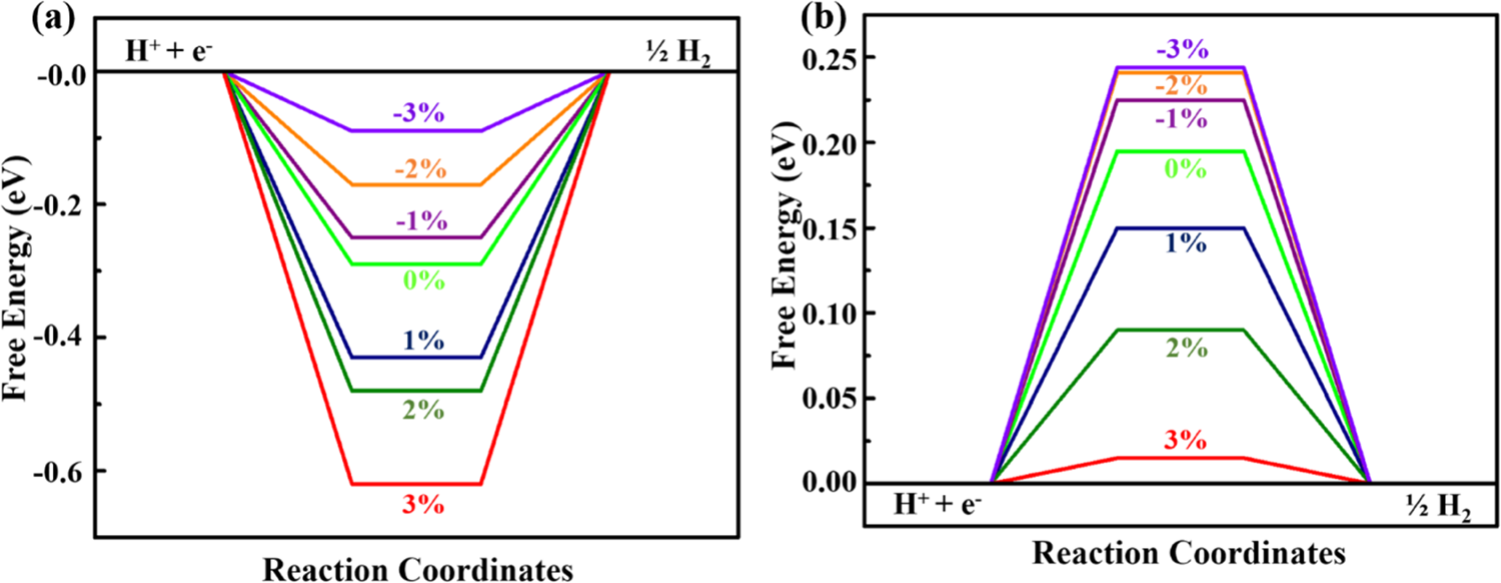
Gibbs free energy diagram of (a) VGe_2_N_4_ and (b) NbGe_2_N_4_ monolayers at different strains ranging from −3 to +3%. Here 0% strain indicates the pristine case.

The biaxial strains (−3 to +3%) are studied to shift the *d*-band center to possess the optimal rate for HER^59^ and observe the change in Δ*G*_H*_. The application of biaxial strain changes the strength of bonding between hydrogen and the substrate. As a result, Δ*G*_H*_ value changes with respect to strain. In both materials, when we increase the strength of the tensile strain, the binding energy of H with monolayers increases and the interatomic distance between the atoms of the monolayer increases. As a result, the interatomic forces between them decrease. Hence, electrons of the monolayer are more prone to bond with H atoms and increase their strength. After analyzing the DOS, we have found the Fermi level to shift toward lower energy with the increase in the strength of the tensile strain, which is shown in Figure 6. This may be the reason for the change in H binding energy with respect to strain. The charge density, density of states, band structures, and band gaps of the two materials under strain are given in the Supporting Information (see Figures S5–S9 and Table S3). For the VGe_2_N_4_ monolayer, Δ*G*_H*_ reduces with compressive strain (Figure 4a). At −3.0% strain, the Δ*G*_H*_ value is −0.09 eV for the hydrogen coverage of 1/4, which indicates that the VGe_2_N_4_ monolayer shows better catalytic performance under compressive strain. The Δ*G*_H*_ values are −0.17, −0.25, −0.43, −0.48, and −0.62 eV under −2, −1, 1, 2, and 3% strains, respectively. By gradually increasing the strains from −3 to +3%, the value of Δ*G*_H*_ is driven toward a higher negative value, which implies the tight binding of the hydrogen atom with tensile strain. However, for the NbGe_2_N_4_ monolayer, the scenario is reversed. For 1/4 hydrogen coverage, the Δ*G*_H*_ reaches zero with the increasing tensile strain (Figure 4b). At 2 and 3% strain, the Δ*G*_H*_ values are determined to be 0.090 and 0.015 eV, respectively, which reveals that the NbGe_2_N_4_ monolayer can be a promising electrocatalyst toward HER under tensile strain. The Δ*G*_H*_ values are 0.150, 0.220, 0.241, and 0.242 eV under 1, −1, −2, and −3% strain respectively. In both cases, applying tensile strain increases the bonding between H and the substrate and compressive strain weakens the bonding. Figure 4 shows that with the same range of biaxial strain, Δ*G*_H*_ changes more for VGe_2_N_4_ than for NbGe_2_N_4_.

**Figure 5:**
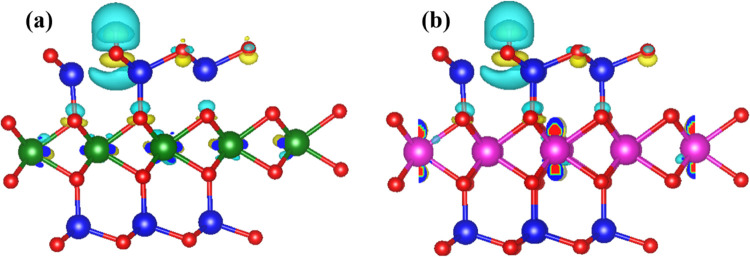
Charge density difference plot for H adsorption on (a) VGe_2_N_4_ and (b) NbGe_2_N_4_ monolayers at 0% strain. (The isosurface value is 0.005 e/A^3^.) Yellow and cyan colors represent electron accumulation and depletion regions, respectively.

**Figure 6:**
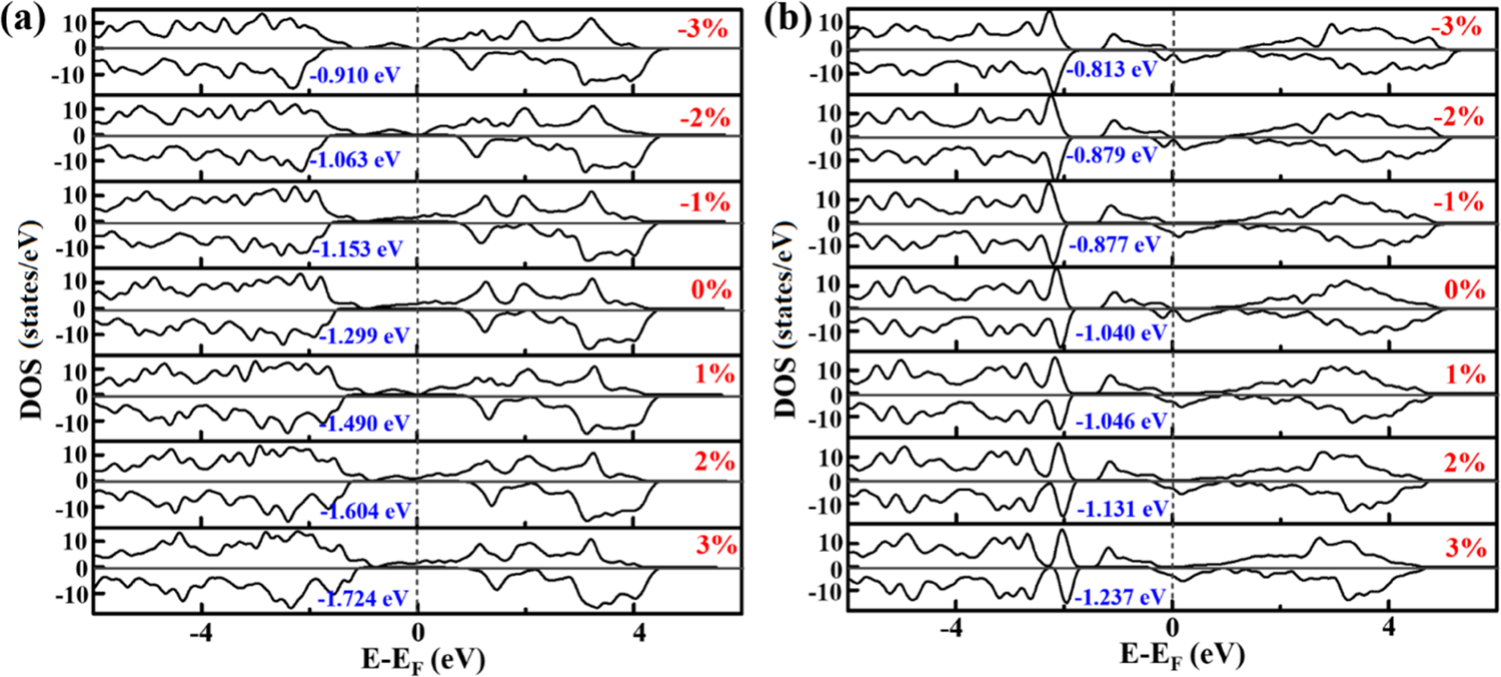
Calculated density of states of (a) VGe_2_N_4_ and (b) NbGe_2_N_4_ monolayers with adsorbed hydrogen at different strains (shown in red color). The gray dashed vertical line represents the Fermi level.

The calculated charge density of the interface between the hydrogen atom and the monolayers is presented for the pristine sample (Figure 5) and density of states with adsorbed hydrogen at different compressive and tensile strains (Figure 6). Furthermore, Bader charge analysis^60^ is used to calculate the charge transfer. Figure 5 shows a significant charge loss from hydrogen towards nitrogen atom for both monolayers, which amounts to 0.48 *e* and 0.42 *e* for VGe_2_N_4_ and NbGe_2_N_4_ monolayers, respectively. In addition, the V and Nb atoms gain some charge (Figure 5a,b). The amount of charge transfer (Δ*Q*) for adsorbed hydrogen atoms on the monolayers at different strains is shown in Table 1. At −3% strain, the charge accumulates at V atoms in the VGe_2_N_4_ monolayer, which shows better HER activity (Figure S5). However, for the NbGe_2_N_4_ monolayer, at +3% strain, very few charges accumulate at Nb atoms. Thus, the accumulation or depletion of charge near the metal atoms plays an active role in HER performance. The variation of binding energies of hydrogen to the monolayers under strains is explained by the density of states (Figure 6). From compressive to tensile strains, the downward shift in the Fermi level for both cases is responsible for the change in HER activity under biaxial strain.

**Table 1:** Calculated Charge Transfer (Δ*Q*) for Adsorbed Hydrogen Atom on Both Monolayers at Different Strains Using Bader Charge Analysis

strain (%)	–3	–2	–1	0	1	2	3	
VGe_2_N_4_	0.478	0.459	0.512	0.481	0.472	0.442	0.427	Δ*Q* (e)
NbGe_2_N_4_	0.447	0.478	0.448	0.421	0.471	0.437	0.412	

Although the accumulated charges on the metal atoms are very low, their existence is clearly shown in Figure 5. As compared to V, a larger charge is accumulated in Nb atoms, which signifies that inner metal atoms also play a role in hydrogen bonding. Thus, we obtain different binding energies for the different monolayers, although the outer-most atoms (Ge and N) of both the layers are the same. In addition, under strain, the charge accumulation near the metal atoms also changes, indicating the affect of metal atoms on HER (Figure S5).

## Conclusions

In summary, we have systematically investigated the catalytic properties of VGe_2_N_4_ and NbGe_2_N_4_ monolayers toward the hydrogen evolution reaction and found that the Gibbs free energy Δ*G*_H*_ is −0.29 and 0.19 eV, respectively, for the pristine VGe_2_N_4_ and NbGe_2_N_4_ monolayers. P doping at the N site in NbGe_2_N_4_ yields a higher Gibbs free energy (Δ*G*_H*_ = 0.11 eV). The strain has a dramatic affect on HER performance on both VGe_2_N_4_ and NbGe_2_N_4_ monolayers. The values of Δ*G*_H*_ of −0.09 eV for VGe_2_N_4_ at −3% strain and 0.015 eV for NbGe_2_N_4_ at +3% strain imply the potential applications of both the monolayers as efficient electrocatalysts for hydrogen production. In addition, compressive strains (−1 to −3%) positively affect the HER activity of the VGe_2_N_4_ monolayer, which weakens the bonding between hydrogen and the substrate, resulting in Δ*G*_H*_ being close to zero. However, the opposite trend is found for the NbGe_2_N_4_ monolayer where the tensile strain (1–3%) increases the interaction between hydrogen and the monolayer, leading to a very low Δ*G*_H*_. Thus, our work provides a direction to experimentalists to not only design VGe_2_N_4_ and NbGe_2_N_4_ monolayers as electrocatalysts for HER activity but also to explore other members of the MA_2_Z_4_ family.

## Supporting Information Available

PBE band structure of VGe_2_N_4_ and NbGe_2_N_4_ monolayers; HSE band structure of VGe_2_N_4_ and NbGe_2_N_4_ monolayers; the values of band gaps for VGe_2_N_4_ and NbGe_2_N_4_ monolayers for different *U* and HSE functionals; Δ*G*_H*_ for N vacancy VGe_2_N_4_ surface doped by different atoms; charge density difference plots for VGe_2_N_4_ and NbGe_2_N_4_ monolayers at different strains; total density of states (DOS) of the VGe_2_N_4_ monolayer under biaxial strains; band structures of the VGe_2_N_4_ monolayer under biaxial strains; band structures of the NbGe_2_N_4_ monolayer under biaxial strains; total density of states (DOS) of the NbGe_2_N_4_ monolayer under biaxial strains; the values of band gaps for VGe_2_N_4_ and NbGe_2_N_4_ monolayers for different biaxial strains; structural, electronic, and catalytic properties of P-doped NbGe_2_N_4_; optimized structure of the P-doped NbGe_2_N_4_ monolayer; the charge density difference for H-adsorbed-doped NbGe_2_N_4_ monolayer; and projected density of states (PDOS) of P doping at N vacancy of the NbGe_2_N_4_ monolayer (PDF)
