# Supplementary material for: Theoretical Insights into the Hydrogen Evolution Reaction on VGe2N4 and NbGe2N4 Monolayers
Source: ACS Omega. 2022 Feb 24;7(9):7837–44. doi: 10.1021/acsomega.1c06730 (PMC8908508; doi:10.1021/acsomega.1c06730)
Supplement: Supplementary file 1 — ao1c06730_si_001.pdf [file ao1c06730_si_001.pdf]

Supporting Information

Theoretical Insights into the Hydrogen Evolution Reaction on VGe_2N_4 and $NbGe_2N_4$ Monolayers

Mihir Ranjan Sahoo[1], Avijeet Ray[2], Nirpendra Singh[3]*

[1]Harish-Chandra Research Institute, Prayagraj, India-211019

[2] Department of Physics, Indian Institute of Technology Roorkee, Roorkee-247667, India

[3]Departemnt of Physics and Center for catalysis and separation (CeCaS), Khalifa University of Science and Technology, Abu Dhabi 127788, United Arab Emirates

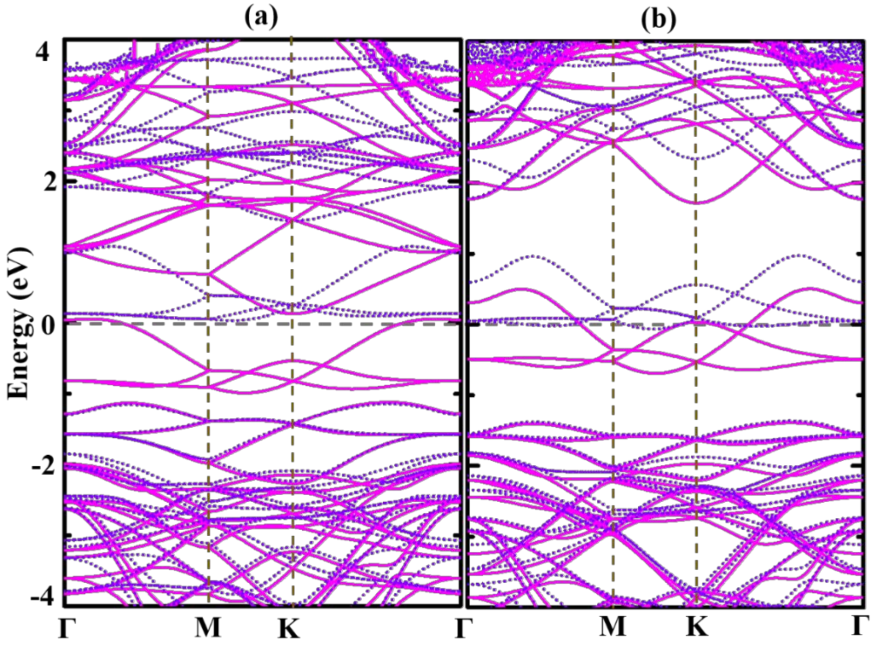

Figure S1 The band structure of (a) VGe_2N_4 and (b) $NbGe_2N_4$ monolayers within PBE functional. Spin-up and spin-down bands are represented by solid magenta and dotted violet lines respectively.

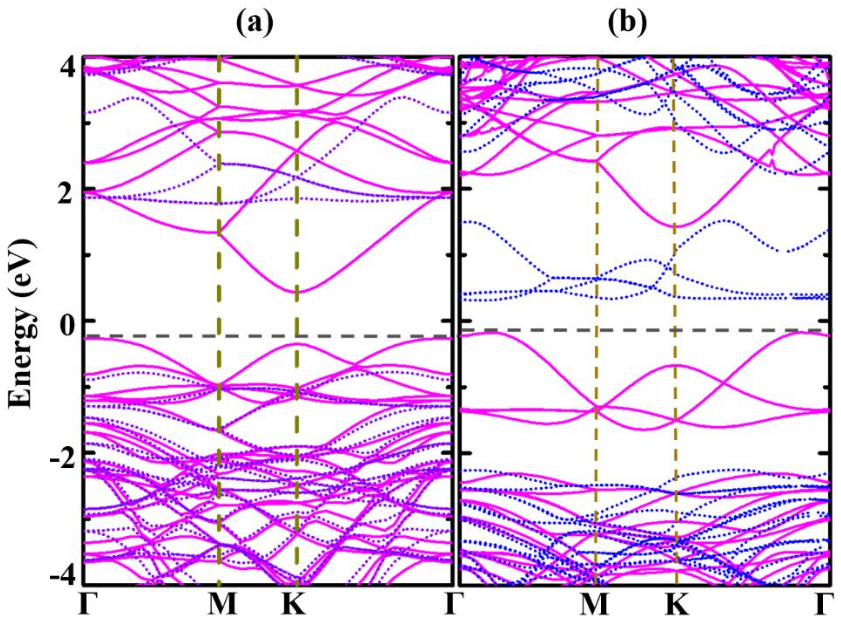

Figure S2 HSE band structure of (a) VGe_2N_4 and (b) $NbGe_2N_4$ monolayers. Spin-up and spin-down bands are represented by solid magenta and dotted violet lines respectively.

Table S1 The values of band gaps for VGe_2N_4 and NbGe$_2$N4 monolayers for different U and HSE functional. Up, down, and total indicate the value for spin-up, spin-down, and total band gaps. For VGe_2N_4 and $NbGe_2N_4$, the HSE total band gap matches with U= 4.5 eV and U= 4 eV respectively.

**U (eV)**	**Band Gap (eV)**					
	**VGe_2N_4**			**$NbGe_2N_4$**		
	Up	Down	Total	Up	Down	Total
2	0.01	1.64	0.01	0.01	1.64	0.01
3	0.02	1.82	0.02	1.15	1.77	0.24
3.5	0.26	1.88	0.26	1.14	1.82	0.33
4	0.49	1.95	0.49	1.12	1.87	0.44
4.5	0.67	2.10	0.67	1.07	1.90	0.55
5	0.63	2.03	0.63	0.89	1.95	0.62
HSE	0.69	2.55	0.69	1.56	2.57	0.48

Table S2 The adsorption Hydrogen Gibbs free energy ΔG_{H*} for N-vacancy VGe_2N_4 surface doped by different atoms.

**Doped atom (X)**	**ΔG_{H*} (eV) at different Position of H**		
	H above N	H above Ge	H above X
**B**	-0.95	-1.60	-1.60
**C**	-1.04	-2.31	-2.31
**O**	0.72	2.46	1.74
**P**	-0.38	1.24	1.29
**S**	0.71	1.30	1.44

Structural, electronic and catalytic properties of P-doped $NbGe_2N_4$

Optimized structure of P atom doped at the N-vacancy of the monolayer is shown in the Figure S3 (a) and (b). The P atom is situated slightly above the outermost layer formed by N atoms. The optimized N-P bond length is found to be 2.27 Å. To check the HER activity of the doped surface in the basal plane, we have considered three different sites for hydrogen adsorption: (i) above N (H_N), (ii) above P (H_P) and (iii) above Ge (H_Ge) atom. Among the three different sites, for H_Ge case, a large positive value for binding energy obtained implying there is no interaction between H and the doped surface. Through Bader, we have found that there is almost no charge transfer between H and surface. Hence we have not proceeded further for details calculations of Ge site. When H is placed above N atom, the binding energy is found to be -0.13 eV whereas for P site, the value is equal to 0.42 eV. Thus ΔG_{H*} values are 0.11 eV and 0.66 eV for N and P sites respectively. This indicates that N site can be considered an active site for HER. The equivalent H-N and H-P bond length was found to be 1.02Å and 1.42Å respectively. To get more insight, we have calculated the amount of charge transfer between H atom and the doped-surface through Bader charge analysis. For case of N-site, a considerable amount of charge transfer (0.49e) from H whereas a small charge (0.28e) is transferred towards H for P-site. The charge density difference diagrams for both the sites are shown in Figure S3 (c) and (d) to understand the charge

redistribution after H adsorption. In case N-site (Figure S3 (c)), a large charge depletion region is observed around H atom and some of the charges are accumulated near the Nb atoms just below it. But the scenario is different in case of P-site as a small amount of charge get accumulated near H indicating very weak interaction (Figure S3 (d)). Further, we have plotted projected density of states (PDOS) of doped monolayer and H-adsorbed above the doped-monolayer (Figure S4) to reveal the electronic structure of H adsorption at different sites. The changes in the DOS near the Fermi level are clearly observed due to H adsorption (Figure S4 (b) and (c)).

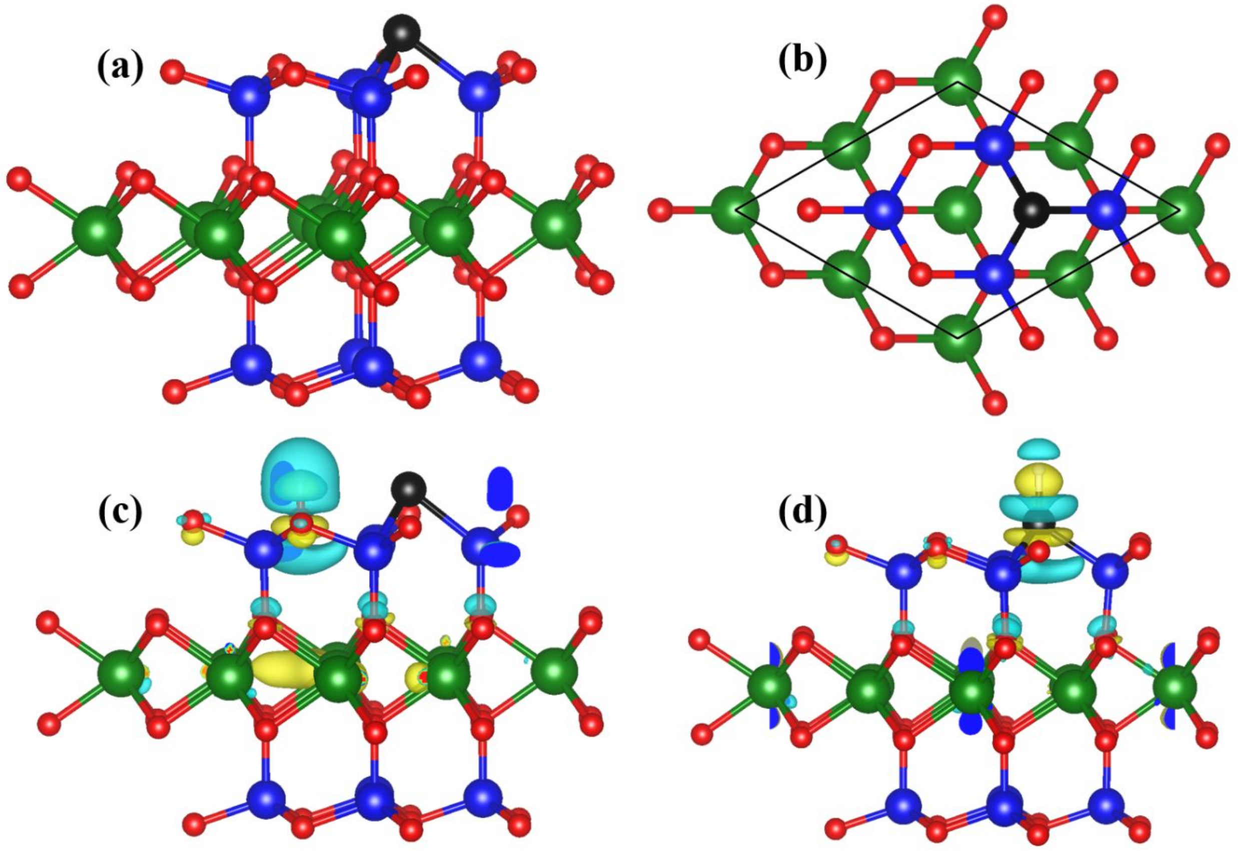

Figure S3 Optimized structure (a) side (b) top view of P-doped $NbGe_2N_4$ monolayer. The charge density difference for (c) H adsorbed above N atom (d) H adsorbed above P atom of the doped monolayer (iso-surface = 0.005 e/Å^3). Nb, Ge, N, C and H atoms are represented by green, blue, red, black and white spheres, respectively. Here P atom is doped in place of N-vacancy of the monolayer. The charge accumulation and depletion regions are represented by yellow and cyan colors, respectively.

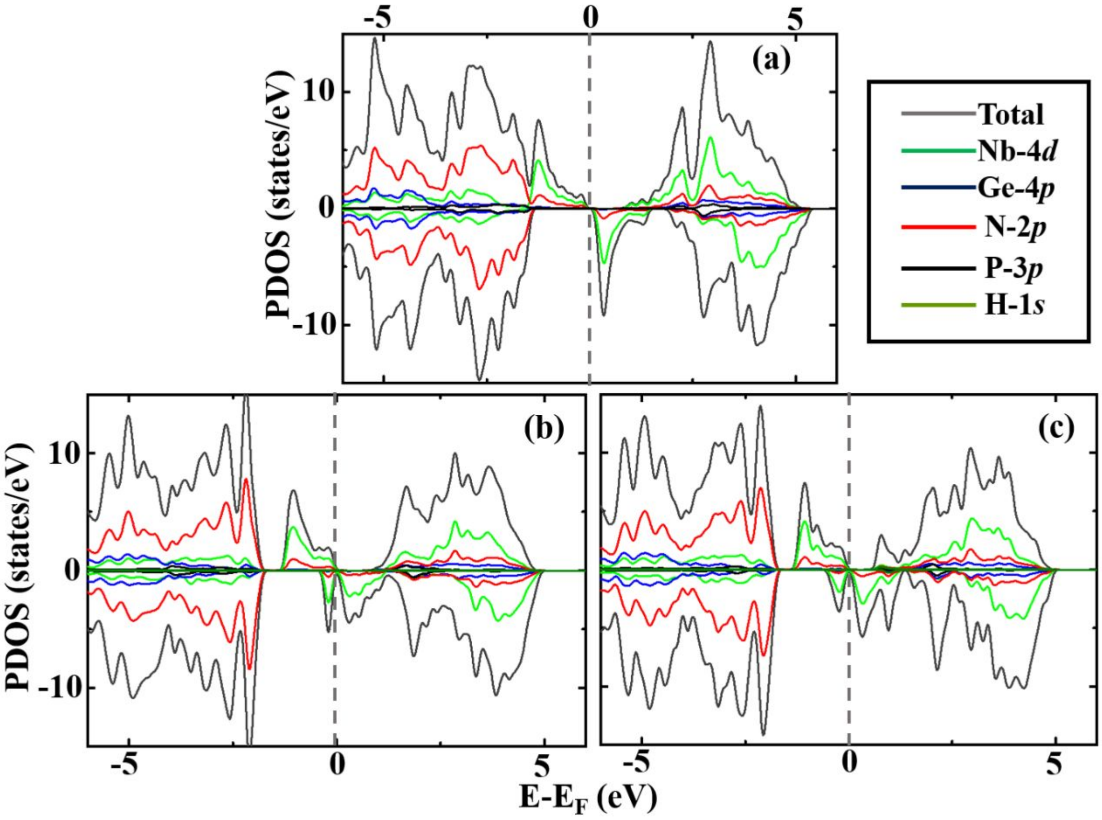

Figure S4 Projected density of states (PDOS) of (a) P-doped at N-vacancy of $NbGe_2N_4$ monolayer (b) H-adsorbed at N-site (c) H-adsorbed at P-site. Fermi level is set at 0 eV.

Electronic structures under biaxial strains:

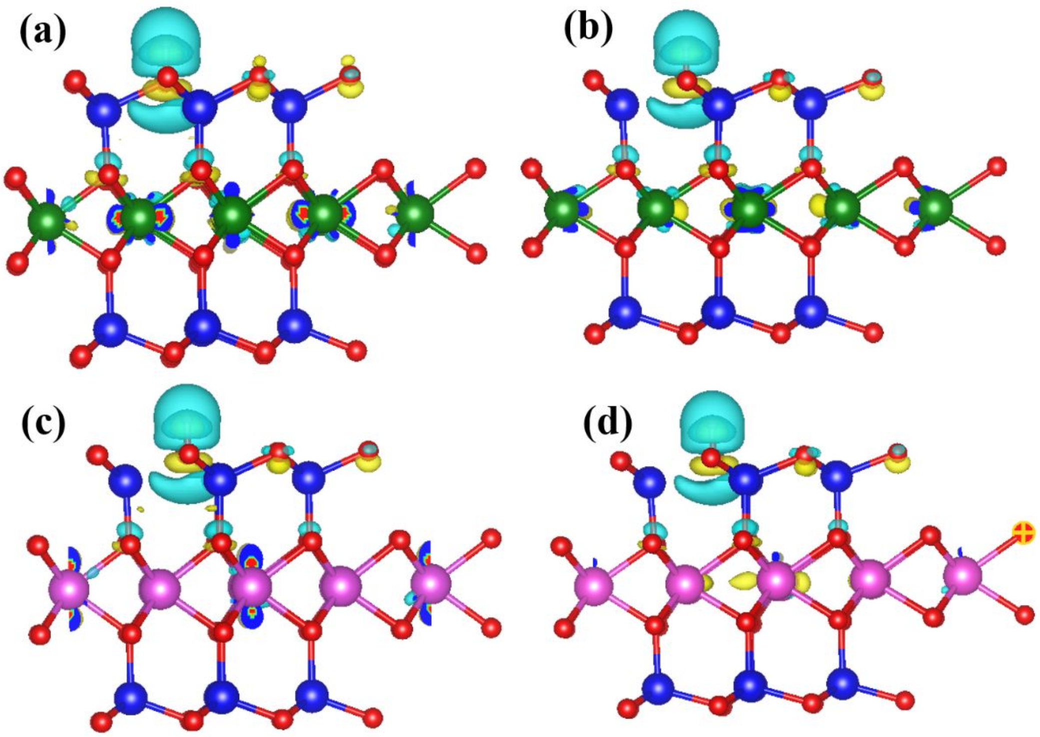

Figure S5 Three-dimensional charge density difference plots for VGe_2N_4 at (a) -3 % (b) +3% strains (upper panel), for $NbGe_2N_4$ monolayers at (c) -3% and (d) +3% strain (lower panel). V, Nb, Ge, and N atoms are represented by green, magenta, blue, and red spheres respectively. (Iso-surface is 0.005 e/A^3). Yellow and cyan color represent electron accumulation and depletion regions respectively.

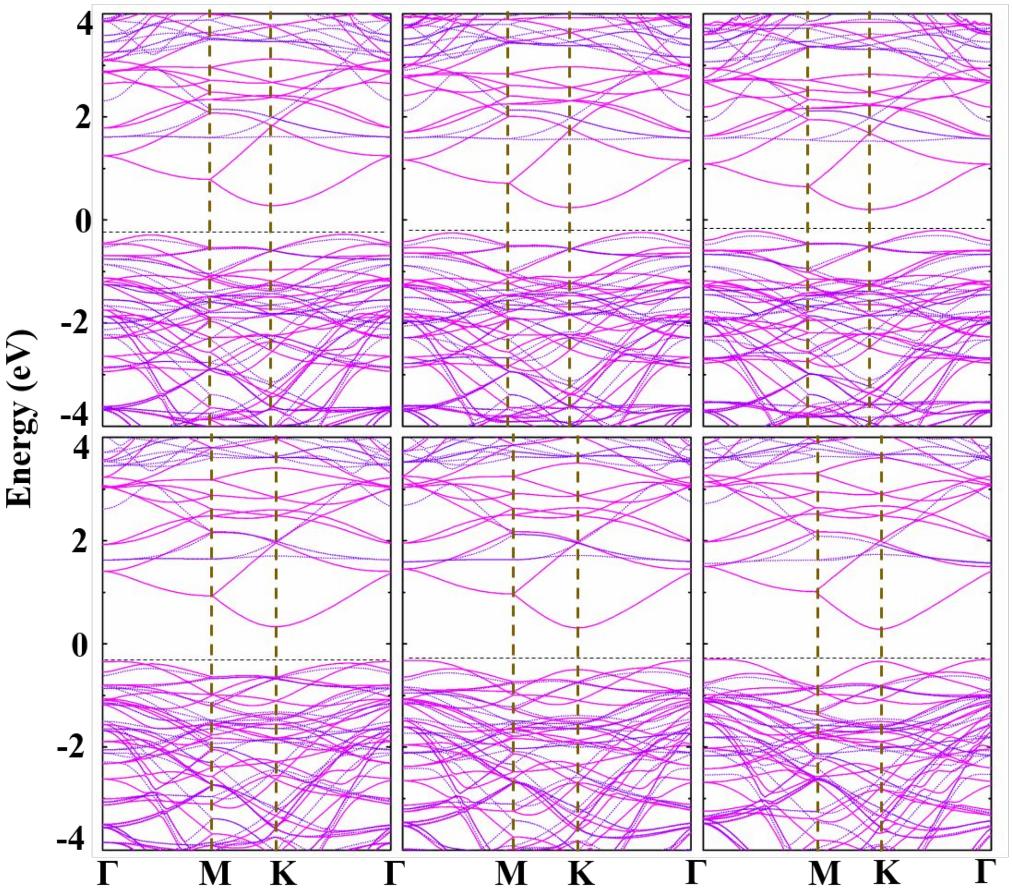

Figure S6 Electronic band structures of VGe_2N_4 monolayer under biaxial strains. Upper panel represents the bands under tensile strains from 1% to 3% while going from left to right. Lower panel represents the bands under compressive strains from -1% to -3% while going from left to right.

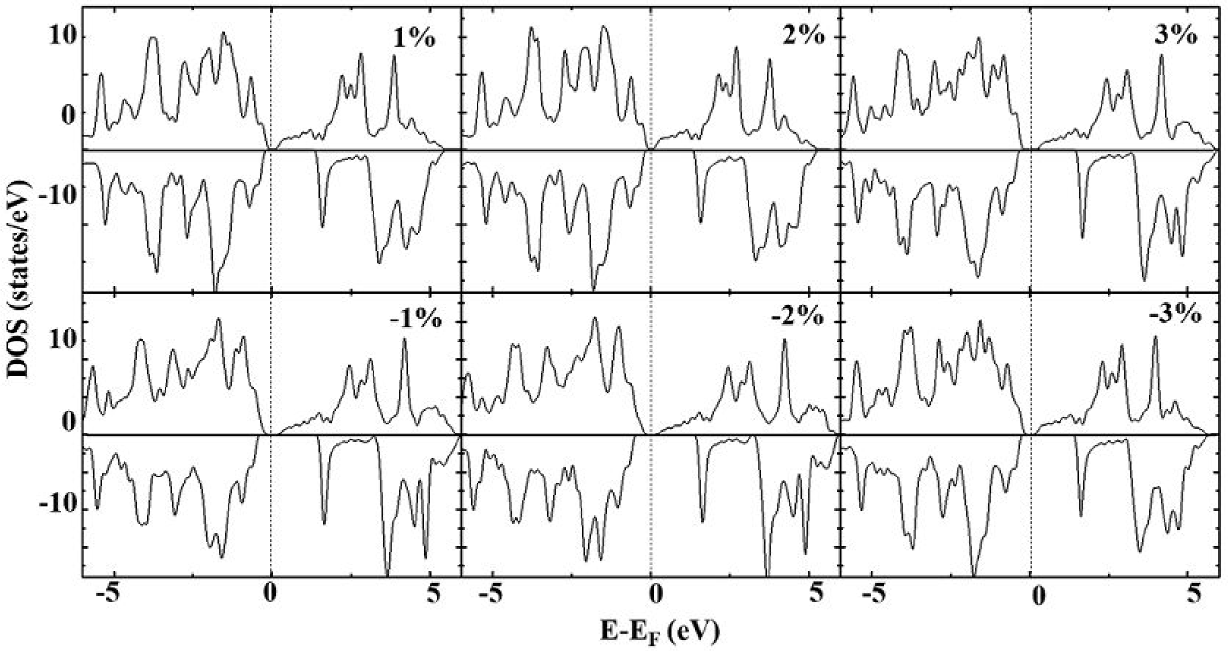

Figure S7 Total density of states (DOS) of VGe_2N_4 monolayer under biaxial strains. Upper panel (tensile strains) and lower panel (compressive strains).

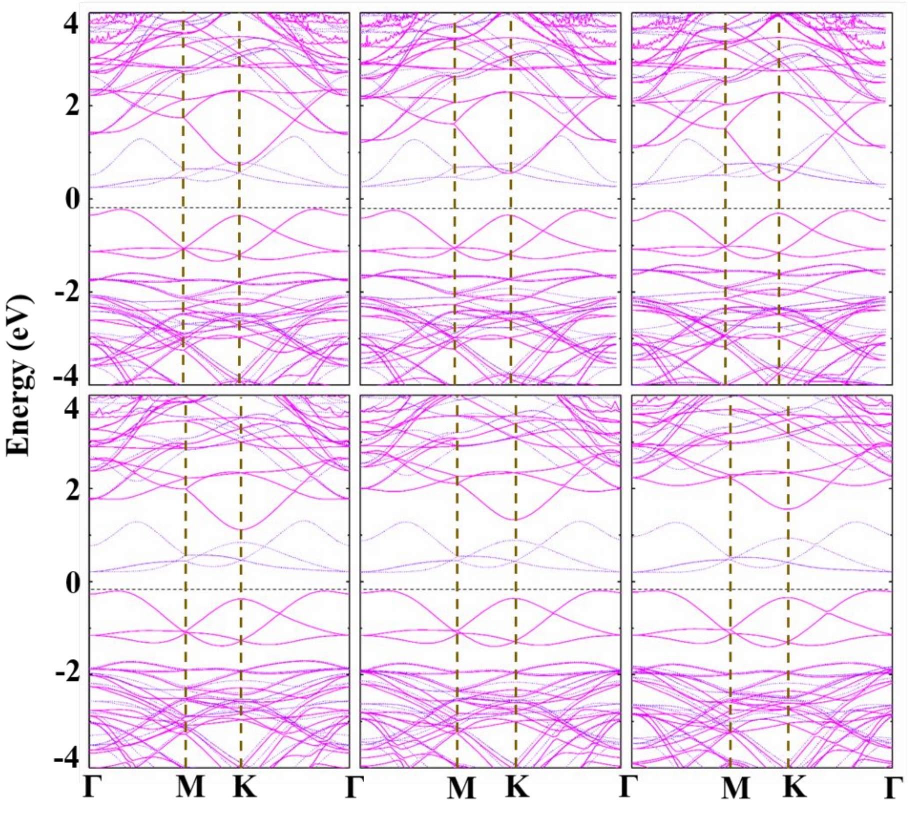

Figure S8 Electronic band structures of $NbGe_2N_4$ monolayer under biaxial strains. Upper panel represents the bands under tensile strains from 1% to 3% while going from left to right. Lower

panel represents the bands under compressive strains from -1% to -3% while going from left to right.

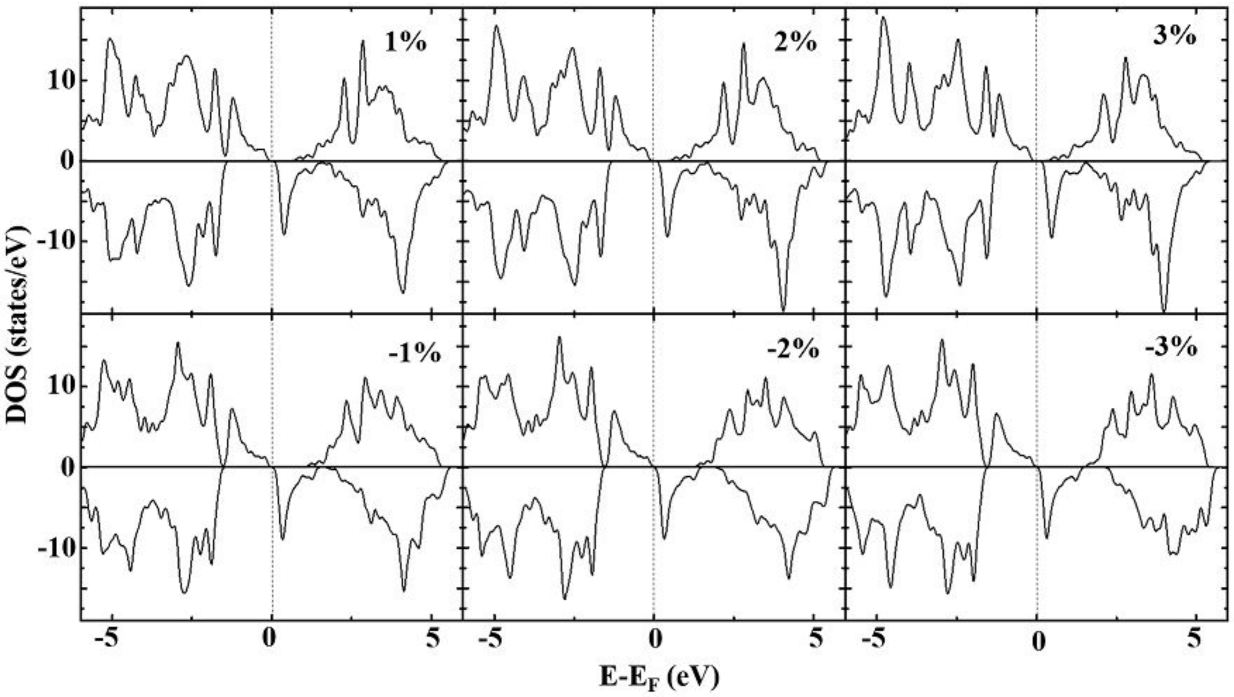

Figure S9 Total density of states (DOS) of $NbGe_2N_4$ monolayer under biaxial strains. Upper panel (tensile strains) and lower panel (compressive strains)

Table S3 The values of band gaps for VGe_2N_4 and $NbGe_2N_4$ monolayers for different biaxial strains. Up, down, and total indicate the value for spin-up, spin-down, and total band gaps.

**Strain (%)**	**Band Gap (eV)**					
	**VGe_2N_4**			**$NbGe_2N_4$**		
	Up	Down	Total	Up	Down	Total
1	0.56	1.96	0.56	0.95	1.82	0.47
2	0.48	1.89	0.48	0.79	1.77	0.51
3	0.40	1.82	0.40	0.63	1.63	0.49
-1	0.67	2.08	0.67	1.31	1.90	0.41
-2	0.63	2.13	0.63	1.52	1.92	0.40
-3	0.58	2.17	0.58	1.74	1.93	0.39
